# Motor and Gait-Based Screening for Low MoCA-BJ Scores in Adults Aged ≥ 50 Years: A Cross-Sectional Study

**DOI:** 10.3390/healthcare14203507

**Published:** 2026-10-19

**Authors:** Lijin Liu, Changfa Huang, Zihua Li, Zhongyin Ji, Yujie Zhou, Xueyi Zhang, Zhihong Wu

**Affiliations:** 1 Stem Cell Facility, Institute of Clinical Medicine, Peking Union Medical College Hospital, Chinese Academy of Medical Sciences and Peking Union Medical College, Beijing 100730, China; liulijin@pumch.cn (L.L.);; 2 Department of Emergency Medicine, Beijing Jishuitan Hospital, Capital Medical University, Beijing 100035, China; 3 Department of Orthopedic Surgery, Peking Union Medical College Hospital, Chinese Academy of Medical Sciences and Peking Union Medical College, Beijing 100730, China; 4 National Infrastructures for Translational Medicine (PUMCH), Institute of Clinical Medicine, Peking Union Medical College Hospital, Chinese Academy of Medical Sciences and Peking Union Medical College, Beijing 100730, China; 5 State Key Laboratory of Complex Severe and Rare Diseases, Peking Union Medical College Hospital, Chinese Academy of Medical Sciences and Peking Union Medical College, Beijing 100730, China

**Keywords:** MoCA, gait, physical performance, cognitive screening, internal validation

## Abstract

**Background/Objectives**: Motor measurements may identify people who merit cognitive assessment, but the added value of detailed gait features is uncertain. We compared simple physical and detailed gait models for classifying Montreal Cognitive Assessment (MoCA) scores at two community sites. **Methods**: After excluding unsuccessful assessments, 371 adults aged ≥50 years with valid MoCA and gait data were included. Five fixed ridge logistic models were evaluated with stratified tenfold cross-validation and fold-specific preprocessing. Paired comparisons, calibration, site validation and threshold analyses were performed. **Results**: MoCA was <26 in 295 participants (79.5%). The parsimonious model had an AUC of 0.707 (0.643 to 0.763); the physical-plus-speed and full-gait models had AUCs of 0.710 and 0.693. Adding detailed gait features to physical measures and speed changed AUC by −0.017 (conditional 95% interval −0.034 to −0.002) and Brier score by +0.0034. Within-site parsimonious AUCs were 0.830 (Pinggu) and 0.565 (Changping). Targeting 90% sensitivity yielded 89.8% sensitivity but 22.4% specificity. **Conclusions**: Simple motor measures moderately discriminated MoCA score groups, and detailed gait features showed no advantage. Performance varied by site, and high sensitivity came with low specificity; harmonized measurement and external validation are the next steps toward screening use.

## 1. Introduction

Earlier recognition of cognitive difficulties can support further assessment in ageing populations. The MoCA samples multiple cognitive domains [1], but a low screening score must be interpreted alongside education, daily functioning and clinical assessment [2,3]. A motor-based model could help prioritize cognitive assessment when physical testing is already part of a community programme. Such a role must be judged by missed cases and additional referrals, as well as by discrimination.

Motor and cognitive functions are related through several pathways. Motoric cognitive risk research links slow gait and cognitive complaints with subsequent adverse outcomes [4,5,6,7], while usual gait speed is a broader marker of functional health [8,9]. Grip strength, reach, chair stands and balance describe complementary physical domains [10,11,12,13,14,15].

Digital systems provide additional information on cadence, gait cycle, stride length, support phases and asymmetry. Prior gait-based cognitive screening and wearable-sensor research support this measurement rationale [16,17,18]. Our question is narrower: whether these additional variables improve concurrent MoCA classification after demographics, simple physical measurements and gait speed are already represented. We therefore compare a fixed hierarchy of models on the same participants and validation splits, including discrimination, prediction error and calibration [19,20,21]. The intended application is to prioritize further cognitive assessment.

## 2. Materials and Methods

### 2.1. Setting and Participants

This cross-sectional analysis used community health-screening records from Pinggu and Changping, Beijing. Screening took place from 18 to 23 August 2025 in Pinggu and from 2 to 4 December 2025 in Changping. Participants were community-dwelling adults who attended these screening events. After duplicate entries were removed, the screening records included 532 individuals. The age threshold of ≥50 years is consistent with the motoric cognitive risk literature [4,5], with a sensitivity analysis restricted to participants aged ≥60 years. Sample size was determined by screening attendance.

Among 467 individuals aged ≥50 years, 32 lacked a successfully acquired MoCA assessment, leaving 435 with valid MoCA. Of these, 64 had no gait record. The final shared model sample comprised 371 participants, including 231 from Pinggu and 140 from Changping.

### 2.2. Cognitive Outcome and Data Quality

Cognitive function was assessed with the Beijing version of the Montreal Cognitive Assessment (MoCA-BJ) at both sites. It was administered and scored by junior rehabilitation therapists trained in MoCA-BJ administration, who did not have access to participants’ motor and gait results. The analyzed total was the sum of the recorded item scores without the education point; recorded totals matched the item sums in all 480 fully scored records. The primary outcome was a recorded MoCA score < 26, with a recorded MoCA score ≥ 26 for the comparison group; the same cutoff was used for all participants. Educational category was available for 112 of 371 participants (Table S4). MoCA < 23 and MMSE < 24 were examined as alternative score definitions.

Nineteen participants with unsuccessful MoCA collection, recorded in the spreadsheet as totals of zero, were excluded. Two Berg records with unsuccessful collection were treated as missing predictors.

Height outside 120–220 cm, weight outside 30–180 kg and derived BMI outside 12–60 kg/m^2^ were set to missing. Variable-level and site-specific missingness, distributions and analysis code are provided in Tables S1 and S2 and File S2.

### 2.3. Measurements and Model Definitions

Both sites used the same model of the Longzhijie GaitWatch inertial three-dimensional gait analysis system. Each walking trial comprised 5 m forward, a turn and 5 m back (10 m total). Participants walked from a standing start without acceleration or deceleration zones, barefoot and without walking aids. Each participant wore seven wireless inertial sensors, placed on the lumbosacral region and bilaterally on the thighs, shanks and feet; the system records spatiotemporal parameters and bilateral hip, knee and ankle joint angles. Gait parameters were exported from the manufacturer’s GaitWatch system software (version 3.0.7.3; Longzhijie Medical Equipment Co., Ltd., Nanjing, China). The software derives speed, stride length and step lengths from the entered test distance, which represents the total distance walked. All values were calculated for the 10 m walked; Pinggu speed, stride length and left and right step lengths, exported with a 5 m setting, were multiplied by 10/5 before analysis. Cadence, cycle time and support-phase percentages do not depend on this setting. Usual-pace walking was performed first and was repeated when the instructions had not been followed; a dual-task walk followed. Usual-pace records were identified by task label in Changping, where they were averaged, and by recording order in Pinggu, where the first valid record, preceding the dual-task walk, was used.

Available physical fields were functional reach in centimetres, repetitions in a 30 s chair-stand test, Berg Balance Scale total and grip strength in kilograms. Grip strength was measured while participants were standing using an Anta electronic dynamometer (Anta Sports, Jinjiang, China). Both hands were tested with three trials per hand; the analytical grip variable was the larger of the left- and right-hand three-trial means, in kilograms. Functional reach was recorded as the maximal forward reach distance while standing (cm), the 30 s chair-stand test as the number of completed stands, and the Berg Balance Scale as the 14-item total (0–56). Gait fields comprised speed (cm/s), cadence (steps/min), cycle time (s), stride length (cm), double support (%), absolute left–right step-length difference (cm) and absolute left–right stance-percentage difference (percentage points). The asymmetry measures were calculated from the participant-level values. Variable coding and derivations are detailed in File S1.

Five prespecified feature sets were compared, without univariate variable selection. The demographic model included age, sex and BMI. The simple physical model added reach, chair stands, Berg score and grip. The physical-plus-speed model added gait speed. The parsimonious model included age, sex, reach, grip and speed. The full-gait model added the six other gait fields to the physical-plus-speed model. The principal incremental comparison was full gait versus physical plus speed, as these two models differ only in the six additional gait fields.

### 2.4. Estimation and Internal Validation

Models minimized summed logistic negative log-likelihood plus one half of the squared non-intercept coefficients (ridge λ = 1). The penalty was fixed and not tuned. The implementation used Newton updates, with convergence checked by the penalized gradient. Every training fold independently supplied predictor medians, means and population standard deviations, which were then applied unchanged to its held-out fold. Binary sex was also standardized for the ridge prediction models. The intercept was not penalized. Final coefficients and preprocessing parameters are given in Table S10.

All models used identical outcome-stratified tenfold partitions (seed 20260627). Performance used pooled out-of-fold (OOF) predictions. We reported AUC, Brier score, calibration-in-the-large with slope fixed at one, and calibration intercept and slope estimated jointly. An intercept-only comparator used the training-fold outcome proportion. Conditional percentile 95% intervals and paired model differences used 2000 outcome-stratified bootstrap samples of the same participant-level OOF records. These intervals describe uncertainty in the evaluation sample; variability from model fitting and fold assignment was examined as described below.

We separately examined partition stability over 30 repeated tenfold partitions. In 300 additional outcome-stratified bootstrap samples, the entire imputation, scaling and model-fitting pipeline was refitted and evaluated on participants absent from that bootstrap sample. Differences between models used the same out-of-bag participants. The resulting percentile ranges describe the robustness of the full pipeline to resampling.

Within-site cross-validation and training on one site followed by testing on the other examined setting dependence. Imputation and scaling used training-site data only. Sensitivity analyses added a site indicator, used complete cases for all candidate predictors, applied alternative cognitive cutoffs and restricted age to ≥60 years. Two further analyses examined gait processing: distance-dependent values without the Pinggu correction, averaged across all Pinggu records, and corrected values averaged across all Pinggu records except the last.

### 2.5. Associations and Threshold Analyses

Associations were estimated separately using unpenalized logistic regression among participants with complete data for age, sex, BMI, reach, grip and speed. Continuous variables were standardized within this complete-case sample; sex remained 0/1, with women as the reference group. Wald intervals used the inverse observed information. A site-adjusted association model was also fitted. Education categories were summarized descriptively because one category had no participants in the MoCA ≥ 26 group. Table 1 uses exploratory Welch tests for continuous variables and a two-sided Fisher exact test for sex.

The Youden threshold from the primary OOF predictions is reported descriptively. To evaluate high-sensitivity thresholds on data not used to select them, an inner cross-validation within each outer training fold selected the highest threshold reaching 90% or 95% sensitivity; the outer-fold model and selected threshold were then evaluated on held-out participants. Decision curves compare score-classification strategies across exploratory probability thresholds [19].

Analyses used Python 3.12.14, NumPy 2.3.5, pandas 2.2.3 and SciPy 1.18.1; plotting used Matplotlib 3.11.2. Reporting was reviewed in relation to TRIPOD+AI and STROBE [20,22]; the completed TRIPOD+AI checklist is provided in Table S11. We used AI tools, including OpenAI Codex (versions 0.154–0.163), to assist with the analysis and language editing, reviewed all content and take full responsibility for it.

## 3. Results

### 3.1. Sample and Missingness

The corrected sample included 371 participants, with a mean age of 64.3 years (SD 9.8); 267 (72.0%) were women. MoCA was <26 in 295 participants (79.5%). Excluding the 19 unsuccessful assessments changed the positive proportion only from 80.5% to 79.5%. Table 1 reports group characteristics with observed denominators. Thirteen participants had at least one missing full-model predictor, leaving 358 complete cases (Table S1). All five primary prediction models used the same 371 participants through training-fold imputation.

### 3.2. Associations

The complete-case association analysis included 362 participants. Per SD, reach was associated with lower odds of MoCA < 26 (OR 0.66, 95% CI 0.48–0.91), as was grip strength (OR 0.64, 0.41–0.98). The speed estimate was 0.77 (0.55–1.09). The male-versus-female OR was 2.83 (1.05–7.68). Full numerical estimates and the site-adjusted model are given in Table S3.

### 3.3. Model Performance and Incremental Value

**Figure 1:**
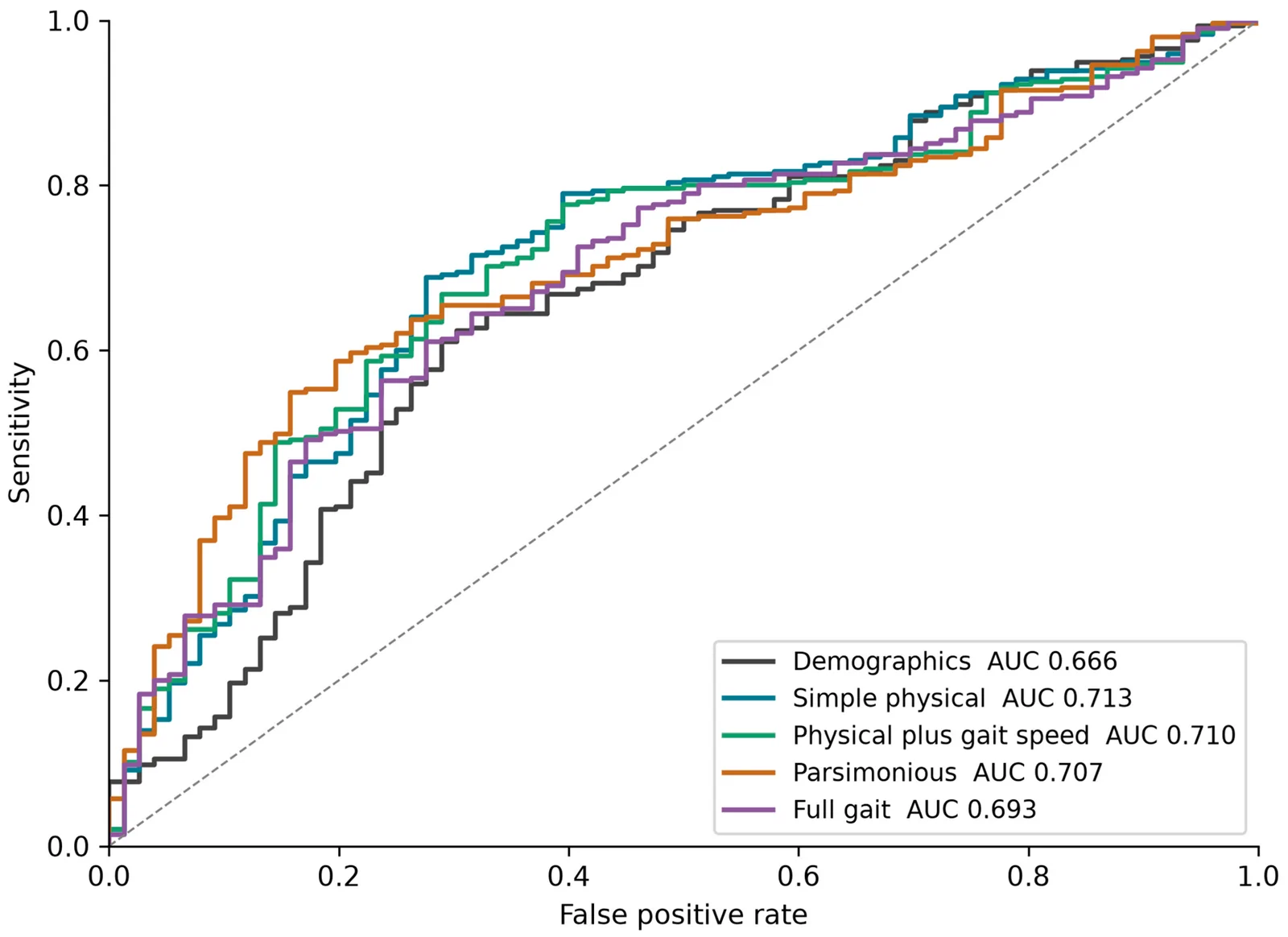
ROC curves from shared out-of-fold predictions in 371 participants. All preprocessing was estimated within each training fold.

The parsimonious model had an AUC of 0.707, compared with 0.713 for simple physical measures, 0.710 after adding speed and 0.693 for the full gait panel (Table 2; Figure 1). Full gait versus physical plus speed gave ΔAUC −0.017 (conditional 95% interval −0.034 to −0.002) and ΔBrier +0.0034 (0.0009 to 0.0063; Table S5). The full panel therefore had slightly lower discrimination and greater prediction error.

**Figure 2:**
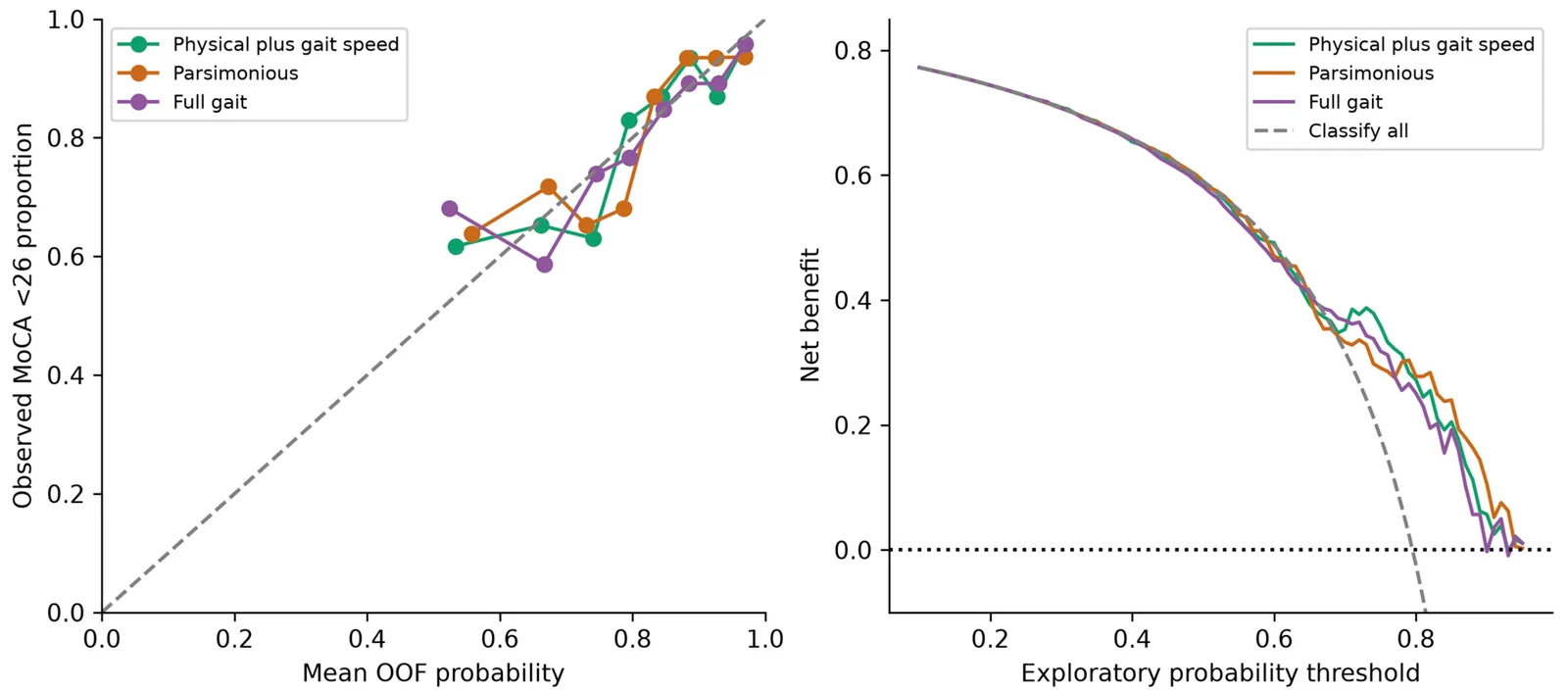
(**Left**): Eight quantile groups of out-of-fold predicted probabilities and observed proportions. (**Right**): Exploratory decision curves for classifying MoCA < 26; the dotted horizontal line denotes classify none.

The intercept-only Brier score was 0.163, compared with 0.149–0.154 for the motor models. The parsimonious calibration slope was 0.874 and the full-gait slope was 0.698; the latter indicated more extreme predicted probabilities relative to observed associations (Figure 2; Table S6). Thirty repeated partitions gave a mean parsimonious AUC of 0.701 (range 0.686–0.710), and ΔAUC for full gait versus physical plus speed was negative in all 30 (mean −0.024). In the full-pipeline out-of-bag analysis, mean ΔAUC for full gait versus physical plus speed was −0.025, with a 2.5th–97.5th percentile range of −0.069 to 0.004 (Table S9).

### 3.4. Site Variation and Sensitivity Analyses

**Figure 3:**
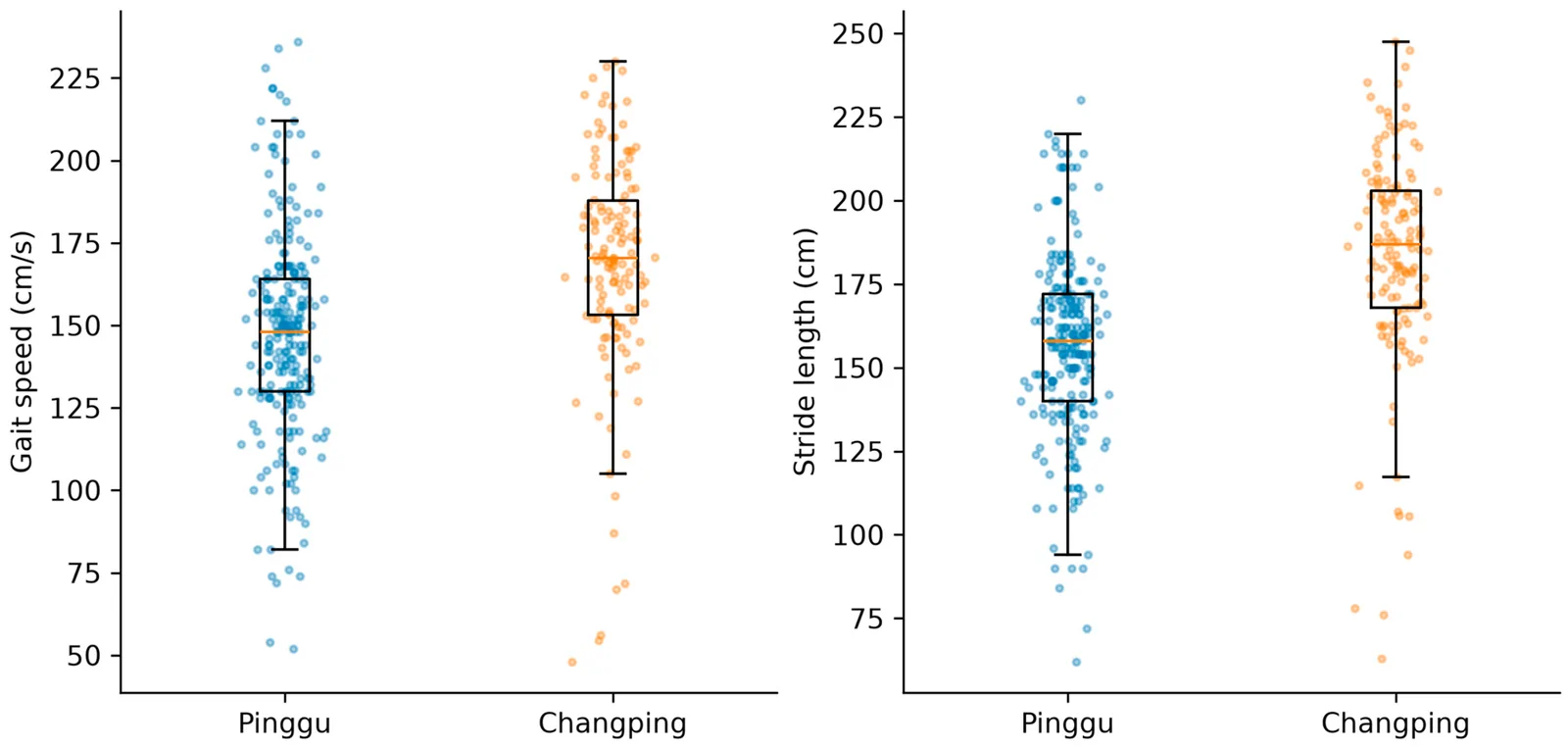
Speed and stride length by site after outcome-data and distance-setting correction. Boxes show quartiles and medians; points show participants.

Speed and stride length were higher in Changping than in Pinggu (medians 170.3 vs. 148.0 cm/s and 186.8 vs. 158.0 cm; Figure 3; Table S2), whereas cadence, gait cycle and double support, which do not depend on the distance setting, were closely comparable (medians 113.0 vs. 111.0 steps/min, 1.06 vs. 1.08 s and 20.0% vs. 20.7% in Pinggu and Changping). Within-site parsimonious AUCs were 0.830 in Pinggu and 0.565 in Changping (Table S8). Training in Changping and testing in Pinggu gave an AUC of 0.719; training in Pinggu and testing in Changping gave 0.551. Adding a site indicator gave a pooled parsimonious AUC of 0.717 (Table S7); site-adjusted association estimates are given in Table S3. For full gait versus physical plus speed, ΔAUC was −0.012 (−0.026 to 0.001) without the distance correction and −0.017 (−0.034 to −0.002) when all Pinggu records except the last were averaged (Table S7).

For the parsimonious model, the AUC was 0.746 for MoCA < 23 (*n* = 371), 0.753 for MMSE <24 (*n* = 336), 0.730 among participants aged ≥60 years (*n* = 227) and 0.714 in complete cases across all candidate predictors (*n* = 358; Table S7). In the 112-person education subset, MoCA < 26 occurred in 15/15 with primary education, 23/27 with junior secondary education, 22/30 with senior secondary education and 21/40 with university education (Table S4). The subset was overwhelmingly from Changping.

### 3.5. Screening Thresholds

At the descriptive Youden threshold, the parsimonious model had a sensitivity of 54.9% and a specificity of 84.2%, missing 133 of 295 participants with MoCA < 26. Nested selection targeting 90% training sensitivity achieved an outer-fold sensitivity of 89.8% and a specificity of 22.4%, with 30 false negatives. Targeting 95% achieved a sensitivity of 95.6% and a specificity of 11.8%, with 13 false negatives. These results indicate that higher sensitivity would result in referrals for most participants without low MoCA scores as well.

## 4. Discussion

Brief motor measurements moderately discriminated MoCA score groups in two community screening samples. In the main incremental comparison, the detailed gait panel had slightly lower AUC and greater prediction error than the smaller physical-plus-speed model; the additional gait features added no information beyond physical measures and gait speed.

The primary contribution is a comparison of additional gait information conditional on simpler measured features, together with an examination of data quality and site transfer. Previous gait-based screening and wearable studies address related measurement questions [16,17,18], and their AUCs reflect different outcomes, devices, populations and validation procedures. Longitudinal motoric cognitive risk evidence [4,5,6,7] also concerns a different endpoint from concurrent MoCA score classification.

Discrimination differed between sites, and demographic models alone showed a similar contrast, indicating that participant composition contributes to it. The principal comparison was consistent within sites, in site-adjusted models and without the Pinggu distance correction, and distance-independent gait measures were closely comparable between sites. Cross-site validation addressed transfer between settings directly.

The 79.5% proportion with MoCA < 26 reflects this screening sample and a uniform cutoff applied without the education point. Education strongly influences MoCA performance in Chinese community settings [23], and the education subset showed a steep gradient. Education-corrected scoring would lower the positive proportion among participants with ≤12 years of education.

Screening value also depends on how many people would be referred. At high sensitivity, outer-fold specificity was low, so most participants with MoCA ≥ 26 would also be referred. Comparing test time, staffing and costs with direct brief cognitive testing is a next step for implementation studies. Motor measurements also support balance, strength and frailty assessment [24,25,26], an area addressed by intervention research [27,28].

This study has limitations. It is cross-sectional, and the 76-person MoCA ≥ 26 group and smaller site samples limit precision. Requiring completed assessments and gait data may have selected participants with better function. Predictor missingness was limited, and complete-case results were similar to those with median imputation. Education, diseases and medications were not comprehensively measured. Validation in an independent cohort is the next step [21,29].

## 5. Conclusions

Simple physical measurements and gait speed classified MoCA score groups with moderate discrimination in these screening records, and additional digital gait features did not improve it. Performance varied between sites, and high sensitivity came with low specificity. Harmonized gait-test settings, education-corrected cognitive scoring and independent validation are the next steps toward community use.

## Figures and Tables

**Table 1:** Characteristics by recorded MoCA score group. Continuous variables are presented as mean (SD), with observed *n* in brackets. *p* values are exploratory and unadjusted.

Variable	MoCA ≥ 26 (*n* = 76)	MoCA < 26 (*n* = 295)	*p*
Age (years)	59.5 (7.4) [76]	65.5 (10.0) [295]	<0.001
Male sex	16/76 (21.1%)	88/295 (29.8%)	0.152
BMI (kg/m^2^)	25.1 (5.4) [76]	25.6 (5.2) [292]	0.518
Functional reach (cm)	27.5 (6.0) [75]	24.0 (6.7) [291]	<0.001
Chair stands (repetitions/30 s)	17.9 (4.7) [76]	15.3 (4.4) [291]	<0.001
Berg balance score	55.0 (2.8) [76]	53.5 (5.5) [293]	<0.001
Grip strength (kg)	27.9 (8.0) [76]	24.9 (8.2) [293]	0.004
Gait speed (cm/s)	168.0 (28.1) [76]	151.9 (35.2) [295]	<0.001
Cadence (steps/min)	113.1 (9.7) [76]	111.3 (10.9) [295]	0.159
Gait cycle (s)	1.1 (0.1) [76]	1.1 (0.1) [295]	0.115
Stride length (cm)	177.9 (24.0) [76]	163.5 (33.6) [295]	<0.001
Double support (%)	20.2 (3.4) [76]	20.8 (3.1) [295]	0.165
Step length asymmetry (cm)	10.1 (8.6) [76]	9.8 (10.8) [295]	0.773
Stance asymmetry (percentage points)	1.6 (1.6) [76]	1.7 (1.9) [295]	0.464
MoCA score	27.3 (1.2) [76]	19.1 (5.0) [295]	Not tested
MMSE score	27.7 (2.1) [69]	24.3 (4.1) [267]	<0.001

**Table 2:** Primary tenfold performance. Values in parentheses following AUCs are conditional 95% bootstrap intervals. Calibration intercept and slope are estimated jointly.

Model	AUC (95% Interval)	Brier	Cal. Intercept	Cal. Slope
Demographics	0.666 (0.594 to 0.734)	0.154	0.174	0.862
Simple physical	0.713 (0.645 to 0.776)	0.149	0.237	0.802
Physical + speed	0.710 (0.641 to 0.769)	0.150	0.250	0.789
Parsimonious	0.707 (0.643 to 0.763)	0.150	0.150	0.874
Full gait	0.693 (0.623 to 0.754)	0.154	0.359	0.698
Intercept only	0.487 (0.424 to 0.555)	0.163	—	—

## Data Availability

The data presented in this study are available on reasonable request from the corresponding author. The data are not publicly available because unrestricted release of individual-level community screening records could compromise participant privacy; access is subject to approval by the relevant ethics committee and institution.

## Supplementary Materials

The following supporting information can be downloaded at: https://www.mdpi.com/article/10.3390/healthcare14203507/s1, Table S1: Missing data by site; Table S2: Site descriptions; Table S3: Association estimates; Table S4: Education subset; Table S5: Paired primary comparisons; Table S6: Calibration; Table S7: Sensitivity analyses; Table S8: Site validation; Table S9: Full pipeline resampling; Table S10: Model coefficients and implementation; Table S11: TRIPOD+AI Reporting Checklist; File S1: Variable definitions and reproducibility; File S2: Code. Participant-level data remain restricted.

## References

[1] Nasreddine Z.S., Phillips N.A., Bédirian V., Charbonneau S., Whitehead V., Collin I., Cummings J.L., Chertkow H. (2005). The Montreal Cognitive Assessment, MoCA: A brief screening tool for mild cognitive impairment. J. Am. Geriatr. Soc..

[2] Atri A., Dickerson B.C., Clevenger C., Karlawish J., Knopman D., Lin P.J., Norman M., Onyike C., Sano M., Scanland S. (2025). Alzheimer’s Association clinical practice guideline for the Diagnostic Evaluation, Testing, Counseling, and Disclosure of Suspected Alzheimer’s Disease and Related Disorders (DETeCD-ADRD): Executive summary of recommendations for primary care. Alzheimers Dement..

[3] Livingston G., Huntley J., Liu K.Y., Costafreda S.G., Selbæk G., Alladi S., Ames D., Banerjee S., Burns A., Brayne C. (2024). Dementia prevention, intervention, and care: 2024 report of the Lancet standing Commission. Lancet.

[4] Verghese J., Annweiler C., Ayers E., Barzilai N., Beauchet O., Bennett D.A., Bridenbaugh S.A., Buchman A.S., Callisaya M.L., Camicioli R. (2014). Motoric cognitive risk syndrome: Multicountry prevalence and dementia risk. Neurology.

[5] Xu W., Bai A., Liang Y., Lin Z. (2024). Motoric Cognitive Risk Syndrome and the Risk of Incident Dementia: A Systematic Review and Meta-Analysis of Cohort Studies. Gerontology.

[6] Lim N.E.-K., Yeo B.S.Y., Lee R.S., Lim J.X., Chan Y.H., Kandiah N., Ho R., Ho C.S.H., Woo J., Arai H. (2024). Motoric cognitive risk syndrome as a predictive factor of cognitive impairment and dementia—A systematic review and meta-analysis. Ageing Res. Rev..

[7] Huang C., Wu B., Zhang C., Wei Z., Su L., Zhang J., Wang L. (2024). Motoric Cognitive Risk Syndrome as a Predictor of Adverse Health Outcomes: A Systematic Review and Meta-Analysis. Gerontology.

[8] Middleton A., Fritz S.L., Lusardi M. (2015). Walking speed: The functional vital sign. J. Aging Phys. Act..

[9] Studenski S., Perera S., Patel K., Rosano C., Faulkner K., Inzitari M., Brach J., Chandler J., Cawthon P., Connor E.B. (2011). Gait speed and survival in older adults. JAMA.

[10] Younis M., Vints W.A.J., Bautmans I. (2026). Handgrip performance as a predictor of incident cognitive decline in older adults: A systematic review and meta-analysis. Neurosci. Biobehav. Rev..

[11] Kobayashi-Cuya K.E., Sakurai R., Suzuki H., Ogawa S., Takebayashi T., Fujiwara Y. (2018). Observational Evidence of the Association Between Handgrip Strength, Hand Dexterity, and Cognitive Performance in Community-Dwelling Older Adults: A Systematic Review. J. Epidemiol..

[12] Neuls P.D., Clark T.L., Van Heuklon N.C., Proctor J.E., Kilker B.J., Bieber M.E., Donlan A.V., Carr-Jules S.A., Neidel W.H., Newton R.A. (2011). Usefulness of the Berg Balance Scale to predict falls in the elderly. J. Geriatr. Phys. Ther..

[13] Low D.C., Walsh G.S. (2022). The minimal important change for measures of balance and postural control in older adults: A systematic review. Age Ageing.

[14] Duncan P.W., Weiner D.K., Chandler J., Studenski S. (1990). Functional reach: A new clinical measure of balance. J. Gerontol..

[15] Rikli R.E., Jones C.J. (2013). Development and validation of criterion-referenced clinically relevant fitness standards for maintaining physical independence in later years. Gerontologist.

[16] Perez-Lasierra J.L., Azpíroz-Puente M., Morita-Hernandez M., Gómez-Bernal A., Alfaro-Santafé J.V., Alfaro-Santafé J. (2025). Gait-Based Screening for Cognitive Impairment in Older Adults: A Fast and Objective Approach. Healthcare.

[17] Zhao H., Cao J., Xie J., Liao W.H., Lei Y., Cao H., Qu Q., Bowen C. (2023). Wearable sensors and features for diagnosis of neurodegenerative diseases: A systematic review. Digit. Health.

[18] Zhao H., Wang R., Qi D., Xie J., Cao J., Liao W.H. (2022). Wearable gait monitoring for diagnosis of neurodegenerative diseases. Measurement.

[19] Vickers A.J., Elkin E.B. (2006). Decision curve analysis: A novel method for evaluating prediction models. Med. Decis. Mak..

[20] Collins G.S., Moons K.G., Dhiman P., Riley R.D., Beam A.L., Van Calster B., Ghassemi M., Liu X., Reitsma J.B., Van Smeden M. (2024). TRIPOD+AI statement: Updated guidance for reporting clinical prediction models that use regression or machine learning methods. BMJ.

[21] Steyerberg E.W., Vickers A.J., Cook N.R., Gerds T., Gonen M., Obuchowski N., Pencina M.J., Kattan M.W. (2010). Assessing the performance of prediction models: A framework for traditional and novel measures. Epidemiology.

[22] von Elm E., Altman D.G., Egger M., Pocock S.J., Gøtzsche P.C., Vandenbroucke J.P., STROBE Initiative (2007). The Strengthening the Reporting of Observational Studies in Epidemiology (STROBE) statement: Guidelines for reporting observational studies. PLoS Med..

[23] Zhou S.A., Zhu J., Zhang N., Wang B., Li T., Lv X., Ng T.P., Yu X., Wang H. (2014). The influence of education on Chinese version of Montreal cognitive assessment in detecting amnesic mild cognitive impairment among older people in a Beijing rural community. Sci. World J..

[24] Fried L.P., Tangen C.M., Walston J., Newman A.B., Hirsch C., Gottdiener J., Seeman T., Tracy R., Kop W.J., Burke G. (2001). Frailty in older adults: Evidence for a phenotype. J. Gerontol. A Biol. Sci. Med. Sci..

[25] Clegg A., Young J., Iliffe S., Rikkert M.O., Rockwood K. (2013). Frailty in elderly people. Lancet.

[26] Sargent L., Brown R. (2017). Assessing the Current State of Cognitive Frailty: Measurement Properties. J. Nutr. Health Aging.

[27] Zeng Z., Ho C.Y., Sit C.H., Wong S.H., Liao J., Yang Y. (2025). Effectiveness of Exercise Intervention on Mobility, Postural Control, and Falls for Older Adults With Mild Cognitive Impairment: A Systematic Review and Network Meta-analysis. Arch. Phys. Med. Rehabil..

[28] Peng J., Chang R., Wei X., Yin Z., Liu Q. (2024). Effect of non-pharmacological interventions in people with cognitive frailty: A systematic review and network meta-analysis. BMC Public Health.

[29] Wolff R.F., Moons K.G., Riley R.D., Whiting P.F., Westwood M., Collins G.S., Reitsma J.B., Kleijnen J., Mallett S., PROBAST Group (2019). PROBAST: A Tool to Assess the Risk of Bias and Applicability of Prediction Model Studies. Ann. Intern. Med..

