# Supplementary material for: Motor and Gait-Based Screening for Low MoCA-BJ Scores in Adults Aged ≥ 50 Years: A Cross-Sectional Study
Source: Healthcare (Basel). 2026 Oct 19;14(20):3507. doi: 10.3390/healthcare14203507 (PMC13703996; doi:10.3390/healthcare14203507)
Supplement: Supplementary file 1 [file healthcare-14-03507-s001.zip › Supplementary_Materials.pdf]

Supplementary Materials: Motor and Gait-Based Screening for Low MoCA-BJ Scores in Adults Aged ≥ 50 Years

Analysis cohort: 371 participants (295 with MoCA <26) from Pinggu and Changping, Beijing. Both sites used the Longzhijie GaitWatch inertial gait analysis system. Cognition was assessed with the MoCA-BJ; no education point was added during data processing or analysis. Pinggu speed, stride length and step lengths were corrected for the software distance setting (File S1).

Table S1 Missing data by site

Variable	Overall	Pinggu	Changping
Age (years)	0/371 (0.0%)	0/231 (0.0%)	0/140 (0.0%)
Male sex	0/371 (0.0%)	0/231 (0.0%)	0/140 (0.0%)
BMI (kg/m²)	3/371 (0.8%)	3/231 (1.3%)	0/140 (0.0%)
Functional reach (cm)	5/371 (1.3%)	4/231 (1.7%)	1/140 (0.7%)
Chair stands (repetitions/30 s)	4/371 (1.1%)	1/231 (0.4%)	3/140 (2.1%)
Berg balance score	2/371 (0.5%)	0/231 (0.0%)	2/140 (1.4%)
Grip strength (kg)	2/371 (0.5%)	1/231 (0.4%)	1/140 (0.7%)
Gait speed (cm/s)	0/371 (0.0%)	0/231 (0.0%)	0/140 (0.0%)
Cadence (steps/min)	0/371 (0.0%)	0/231 (0.0%)	0/140 (0.0%)
Gait cycle (s)	0/371 (0.0%)	0/231 (0.0%)	0/140 (0.0%)
Stride length (cm)	0/371 (0.0%)	0/231 (0.0%)	0/140 (0.0%)
Double support (%)	0/371 (0.0%)	0/231 (0.0%)	0/140 (0.0%)
Step length asymmetry (cm)	0/371 (0.0%)	0/231 (0.0%)	0/140 (0.0%)
Stance asymmetry (percentage points)	0/371 (0.0%)	0/231 (0.0%)	0/140 (0.0%)
MoCA score	0/371 (0.0%)	0/231 (0.0%)	0/140 (0.0%)
MMSE score	35/371 (9.4%)	35/231 (15.2%)	0/140 (0.0%)

MoCA totals of zero produced by wholly unsuccessful assessments were treated as missing outcomes and excluded; wholly unsuccessful Berg assessments were treated as missing predictors. All primary prediction models use the same 371 participants; 358 are complete for all 14 candidate predictors.

Table S2 Site descriptions

Variable	Pinggu mean (SD) [n]	Changping mean (SD) [n]
Age (years)	64.55 (8.41) [231]	63.85 (11.76) [140]
Male sex	0.26 (0.44) [231]	0.31 (0.46) [140]
BMI (kg/m²)	26.56 (5.98) [228]	23.78 (2.91) [140]
Functional reach (cm)	25.18 (6.94) [227]	23.91 (6.32) [139]
Chair stands (repetitions/30 s)	15.23 (4.09) [230]	16.88 (5.11) [137]
Berg balance score	54.59 (3.21) [231]	52.41 (7.07) [138]
Grip strength (kg)	24.82 (8.04) [230]	26.61 (8.53) [139]
Gait speed (cm/s)	147.25 (32.36) [231]	168.40 (33.90) [140]
Cadence (steps/min)	112.68 (10.84) [231]	109.89 (10.13) [140]
Gait cycle (s)	1.08 (0.13) [231]	1.11 (0.13) [140]
Stride length (cm)	156.12 (27.79) [231]	183.45 (32.41) [140]
Double support (%)	20.40 (2.99) [231]	21.11 (3.34) [140]
Step length asymmetry (cm)	10.23 (10.84) [231]	9.14 (9.62) [140]
Stance asymmetry (percentage points)	1.64 (1.70) [231]	1.82 (2.04) [140]
MoCA score	19.66 (6.13) [231]	22.59 (3.95) [140]
MoCA <26, n (%)	192/231 (83.1%)	103/140 (73.6%)
MMSE score	23.90 (4.37) [196]	26.63 (2.73) [140]

Pinggu speed, stride length and step-length asymmetry are shown after the 10/5 distance-setting correction (File S1); no other cross-site calibration or unit conversion was applied. The cohort includes 231 Pinggu and 140 Changping participants.

Table S3 Association estimates

Complete-case association model

Predictor	OR (95% CI)	P
Age (per SD)	1.44 (0.97–2.13)	0.069
Male vs female	2.83 (1.05–7.68)	0.040
BMI (per SD)	1.18 (0.87–1.60)	0.281
Functional reach (per SD)	0.66 (0.48–0.91)	0.011
Grip strength (per SD)	0.64 (0.41–0.98)	0.039
Gait speed (per SD)	0.77 (0.55–1.09)	0.143

Additional adjustment for site

Predictor	OR (95% CI)	P
Age (per SD)	1.43 (0.97–2.10)	0.069
Male vs female	2.88 (1.06–7.86)	0.039
BMI (per SD)	1.12 (0.82–1.53)	0.467
Functional reach (per SD)	0.64 (0.46–0.89)	0.007
Grip strength (per SD)	0.64 (0.42–0.99)	0.044
Gait speed (per SD)	0.83 (0.58–1.20)	0.320
Site: Changping vs Pinggu	0.67 (0.36–1.24)	0.204

Both models use 362 complete cases. Continuous-predictor effects are per SD in the complete-case sample; sex is male versus female, and site is Changping versus Pinggu. Estimates are unpenalized maximum likelihood with Wald intervals; imputed values are not used.

Table S4 Education subset

Education	n	MoCA <26	Mean MoCA
Primary	15	15	18.80
Junior secondary	27	23	21.63
Senior secondary	30	22	22.93
University	40	21	24.40

The analysed MoCA-BJ total is the sum of recorded item scores, without the education point or education-specific thresholds. One conflicting duplicate education identifier was excluded. A zero cell in the primary-education category causes separation, so education-adjusted odds ratios are not reported. The subset comes predominantly from Changping.

Table S5 Paired primary comparisons

Comparison	ΔAUC (conditional 95%)	ΔBrier (conditional 95%)
Full gait minus physical + speed	-0.017 (-0.034 to -0.002)	0.0034 (0.0009 to 0.0063)
Physical + speed minus simple physical	-0.003 (-0.015 to 0.008)	0.0010 (-0.0012 to 0.0033)
Parsimonious minus demographics	0.041 (-0.014 to 0.095)	-0.0035 (-0.0116 to 0.0046)
Full gait minus parsimonious	-0.014 (-0.045 to 0.017)	0.0036 (-0.0015 to 0.0089)

Negative ΔAUC favours the second model; positive ΔBrier means greater error in the first model. Intervals come from 2,000 outcome-stratified resamples of the fixed OOF predictions, using the same indices for both models.

Table S6 Calibration

Model	CITL (conditional 95%)	Slope (conditional 95%)
Demographics	0.004 (-0.067 to 0.072)	0.862 (0.468 to 1.344)
Simple physical	0.004 (-0.089 to 0.099)	0.802 (0.489 to 1.184)
Physical + speed	0.004 (-0.088 to 0.100)	0.789 (0.491 to 1.156)
Parsimonious	0.004 (-0.081 to 0.090)	0.874 (0.560 to 1.229)
Full gait	0.001 (-0.094 to 0.097)	0.698 (0.416 to 1.028)

CITL is calibration-in-the-large with the slope fixed at one; the slope is estimated jointly with an intercept. Joint intercept point estimates are given in manuscript Table 2.

Table S7 Sensitivity analyses

Scenario	n / events	Physical + speed AUC	Parsimonious AUC	Full gait AUC
MoCA <23	371 / 205	0.749	0.746	0.742
MMSE <24	336 / 94	0.780	0.753	0.768
Age ≥60 years	227 / 197	0.695	0.730	0.658
Complete cases	358 / 283	0.716	0.714	0.683
Site indicator added	371 / 295	0.709	0.717	0.692
No distance correction, all Pinggu records averaged	371 / 295	0.715	0.721	0.703
Pinggu records except the last averaged	371 / 295	0.708	0.705	0.691

The primary analysis uses corrected distance-dependent values and the first valid Pinggu record (File S1). For full gait minus physical + speed, ΔAUC was -0.012 (-0.026 to 0.001) and ΔBrier 0.0042 (0.0008 to 0.0084) without the distance correction, and -0.017 (-0.034 to -0.002) and 0.0043 (0.0015 to 0.0075) when all Pinggu records except the last were averaged; intervals are conditional, as in Table S5.

Table S8 Site validation

Test site and procedure	n	Physical + speed AUC	Parsimonious AUC	Full gait AUC
Pinggu, within-site CV	231	0.812	0.830	0.794
Pinggu, trained on Changping	231	0.668	0.719	0.607
Changping, within-site CV	140	0.561	0.565	0.504
Changping, trained on Pinggu	140	0.560	0.551	0.558

When training on one site and testing on the other, imputation and scaling parameters come from the training site only. All analyses use the 371-participant cohort.

Table S9 Full pipeline resampling

Model	Mean OOB AUC	2.5th–97.5th percentile
Demographics	0.664	0.585–0.737
Simple physical	0.700	0.621–0.787
Physical + speed	0.692	0.611–0.777
Parsimonious	0.697	0.622–0.770
Full gait	0.668	0.581–0.750
Full gait minus physical + speed (ΔAUC)	-0.025	-0.069–0.004

Each of 300 bootstrap samples refits all preprocessing and model coefficients and is evaluated on out-of-bag participants only. The percentile ranges describe resampling variability of the full pipeline. Repeated tenfold AUC results are summarised in Section 3.3 of the manuscript.

Table S10 Model coefficients and implementation

For each model, missing predictors are replaced by the training median and each predictor is standardized as $z = (x - \text{mean})/\text{SD}$; the predicted probability is $p = 1/(1 + \exp(-(\text{intercept} + \Sigma\beta z)))$. The file final_coefficients_research_only.json (File S2) gives the intercepts, coefficients, medians, means and SDs for all five models. The coefficients are provided for research reproduction.

The table below shows the parsimonious model (standardized intercept 1.631127).

Predictor	Median	Mean	SD	Coefficient
Age, years [age]	63.00000	64.28841	9.78504	0.376094
Male sex [male]	0.00000	0.28032	0.44916	0.433386
Functional reach, cm [frt_cm]	25.00000	24.70081	6.67412	-0.366290
Grip strength, kg [grip_max_avg]	24.53333	25.48702	8.23288	-0.429176
Gait speed, cm/s [gait_speed_cms]	155.00000	155.22911	34.42167	-0.246927

Table S11 Reporting checklist

The completed TRIPOD+AI checklist is provided as a separate file (Table S11).

File S1 Variable definitions and reproducibility

Sex is coded male=1, female=0. Site indicator is Changping=1, Pinggu=0. Grip is the larger of two hand-specific three-trial means. Absolute left–right step-length and stance-percentage differences are computed from participant-level values. Other variables retain the exported units shown in Tables S1–S2. Both sites used the same Longzhijie GaitWatch model, with seven inertial sensors (lumbosacral region and bilateral thighs, shanks and feet); each walking trial was a 5 m outward and 5 m return walk (10 m total) without acceleration or deceleration buffers, footwear or walking aids. The software derives speed, stride length and step lengths from the entered test distance, which represents the total distance walked. All values were calculated for the 10 m walked; Pinggu speed, stride length and left and right step lengths, exported with a 5 m setting, were multiplied by 10/5. Cadence, gait cycle and double-support percentage do not depend on this setting. Usual-pace walking preceded a dual-task walk. Usual-pace records were identified by task label in Changping (averaged) and by recording order in Pinggu (first valid record). In the labelled Changping data, every dual-task record followed all usual-pace records, and dual-task speed was a median 0.91 times usual-pace speed; the last Pinggu record showed the same pattern (median 0.91 times the earlier records), whereas repeated usual-pace records did not (Changping 1.03). Sensitivity analyses using uncorrected values and averaging all Pinggu records except the last (a single record was kept as recorded) are given in Table S7.

Analyses can be reproduced with the scripts in File S2 (see README_code.md); verify_trial_order.py reproduces the trial-order check. The source workbook is not shared. Raw-file SHA256 and package versions are recorded in provenance.json. This supplement contains no participant-level data.
