# Supplementary material for: Motor and Gait-Based Screening for Low MoCA-BJ Scores in Adults Aged ≥ 50 Years: A Cross-Sectional Study
Source: Healthcare (Basel). 2026 Oct 19;14(20):3507. doi: 10.3390/healthcare14203507 (PMC13703996; doi:10.3390/healthcare14203507)
Supplement: Supplementary file 1 [file healthcare-14-03507-s001.zip › Table S11 TRIPOD_AI_Checklist.pdf]

TRIPOD+AI Reporting Checklist — healthcare-4558735

Version: 11-January-2024. From: Collins GS, Moons KGM, Dhiman P, et al. *BMJ* 2024;385:e078378. doi:10.1136/bmj-2023-078378.

Study type: **Development with internal validation (D)**.

Section/Topic	Item	D/E	Checklist item	Reported on page
**TITLE**				
Title	1	D;E	Identify the study as developing or evaluating the performance of a multivariable prediction model, the target population, and the outcome to be predicted	Title
**ABSTRACT**				
Abstract	2	D;E	See TRIPOD+AI for Abstracts checklist	Abstract
**INTRODUCTION**				
Background	3a	D;E	Explain the healthcare context and rationale for developing or evaluating the prediction model, including	Introduction para 1–2

Section/Topic	Item	D/E	Checklist item	Reported on page
			references to existing models	
	3b	D;E	Describe the target population and the intended purpose of the prediction model in the context of the care pathway	Introduction paras 1 and 3
	3c	D;E	Describe any known health inequalities between sociodemographic groups	Introduction para 1 (education)
Objectives	4	D;E	Specify the study objectives, including whether the study describes the development or validation of a prediction model (or both)	Introduction para 3
**METHODS**				
Data	5a	D;E	Describe the sources of data separately for the development and evaluation datasets, the	Methods 2.1

Section/Topic	Item	D/E	Checklist item	Reported on page
			rationale for using these data, and representativeness of the data	
	5b	D;E	Specify the dates of the collected participant data, including start and end of participant accrual; and, if applicable, end of follow-up	Methods 2.1
Participants	6a	D;E	Specify key elements of the study setting (e.g., primary care, secondary care, general population) including the number and location of centres	Methods 2.1 (two community sites, Pinggu and Changping, Beijing)
	6b	D;E	Describe the eligibility criteria for study participants	Methods 2.1
	6c	D;E	Give details of any treatments received, and how they were handled during model	Not applicable (cross-sectional screening)

Section/Topic	Item	D/E	Checklist item	Reported on page
			development or evaluation, if relevant	
Data preparation	7	D;E	Describe any data pre-processing and quality checking, including whether this was similar across relevant sociodemographic groups	Methods 2.2, 2.3; Table S1; File S1 (including the Pinggu distance-setting correction and usual-pace record selection)
Outcome	8a	D;E	Clearly define the outcome that is being predicted and the time horizon, including how and when assessed, the rationale for choosing this outcome, and whether the method of outcome assessment is consistent across sociodemographic groups	Methods 2.2 (MoCA-BJ <26)
	8b	D;E	If outcome assessment requires subjective interpretation, describe the	Methods 2.2 (junior rehabilitation therapists trained in MoCA-BJ

Section/Topic	Item	D/E	Checklist item	Reported on page
			qualifications and demographic characteristics of the outcome assessors	administration)
	8c	D;E	Report any actions to blind assessment of the outcome to be predicted	Methods 2.2 (MoCA-BJ assessors did not have access to motor and gait results)
Predictors	9a	D	Describe the choice of initial predictors (e.g., literature, previous models, all available predictors) and any pre-selection of predictors before model building	Methods 2.3 (five prespecified feature sets; no univariate selection)
	9b	D;E	Clearly define all predictors, including how and when they were measured (and any actions to blind assessment of predictors for the outcome	Methods 2.3; File S1

Section/Topic	Item	D/E	Checklist item	Reported on page
			and other predictors)	
	9c	D;E	If predictor measurement requires subjective interpretation, describe the qualifications and demographic characteristics of the predictor assessors	Methods 2.3 (gait: automated inertial system; physical-test assessor details not reported)
Sample size	10	D;E	Explain how the study size was arrived at, and justify that the study size was sufficient to answer the research question. Include details of any sample size calculation	Methods 2.1 (sample size determined by screening attendance); Discussion para 6
Missing data	11	D;E	Describe how missing data were handled. Provide reasons for omitting any data	Methods 2.2, 2.4; Table S1 (median imputation within training folds)
Analytical methods	12a	D	Describe how the data were used (e.g., for development and evaluation of model	Methods 2.4 (stratified 10-fold CV with fold-specific preprocessing)

Section/Topic	Item	D/E	Checklist item	Reported on page
			performance) in the analysis, including whether the data were partitioned, considering any sample size requirements	
	12b	D	Depending on the type of model, describe how predictors were handled in the analyses (functional form, rescaling, transformation, or any standardisation)	Methods 2.4 (z-standardisation within each training fold)
	12c	D	Specify the type of model, rationale, all model-building steps, including any hyperparameter tuning, and method for internal validation	Methods 2.4 (ridge logistic regression, L2 penalty λ=1 fixed, stratified 10-fold CV, 300 OOB bootstrap)
	12d	D;E	Describe if and how any heterogeneity in estimates of model parameter	Methods 2.4; Results 3.4; Tables S2 and S8 (within-site and cross-site evaluation)

Section/Topic	Item	D/E	Checklist item	Reported on page
			values and model performance was handled and quantified across clusters	
	12e	D;E	Specify all measures and plots used (and their rationale) to evaluate model performance (e.g., discrimination, calibration, clinical utility) and, if relevant, to compare multiple models	Methods 2.4–2.5 (AUC, Brier, calibration intercept/slope; decision curves in 2.5); Figures 1–2
	12f	E	Model updating (e.g., recalibration) arising from the model evaluation	Not applicable (development study)
	12g	E	For model evaluation, describe how the model predictions were calculated	Not applicable (development study)
Class imbalance	13	D;E	If class imbalance methods were used, state	Not used (no class weighting or resampling;

Section/Topic	Item	D/E	Checklist item	Reported on page
			why and how this was done, and any subsequent methods to recalibrate the model or the model predictions	see analysis code)
Fairness	14	D;E	Describe any approaches that were used to address model fairness and their rationale	Not formally assessed; site and education differences discussed (Discussion paras 3–4)
Model output	15	D	Specify the output of the prediction model (e.g., probabilities, classification). Provide details and rationale for any classification and how the thresholds were identified	Methods 2.5 (predicted probabilities; nested threshold selection; decision curves)
Training vs evaluation	16	D;E	Identify any differences between the development and evaluation data in healthcare setting, eligibility criteria, outcome, and predictors	Methods 2.4 (single cohort evaluated by cross-validation; within-site and cross-site analyses)

Section/Topic	Item	D/E	Checklist item	Reported on page
Ethical approval	17	D;E	Name the institutional research board or ethics committee that approved the study and describe the participant-informed consent or the ethics committee waiver of informed consent	Ethics statement (project No. K8400; acceptance No. K25C2036)
**OPEN SCIENCE**				
Funding	18a	D;E	Give the source of funding and the role of the funders for the present study	Funding statement
Conflicts of interest	18b	D;E	Declare any conflicts of interest and financial disclosures for all authors	Conflicts of Interest statement
Protocol	18c	D;E	Indicate where the study protocol can be accessed or state that a protocol was not prepared	Not reported
Registration	18d	D;E	Provide registration	Not reported

Section/Topic	Item	D/E	Checklist item	Reported on page
			information for the study, or state that the study was not registered	
Data sharing	18e	D;E	Provide details of the availability of the study data	Data Availability Statement
Code sharing	18f	D;E	Provide details of the availability of the analytical code	Methods 2.2; code package in File S2
**PATIENT & PUBLIC INVOLVEMENT**				
Patient & Public Involvement	19	D;E	Provide details of any patient and public involvement during the design, conduct, reporting, interpretation, or dissemination of the study or state no involvement	Not reported
**RESULTS**				
Participants	20a	D;E	Describe the flow of participants through the study, including the number of	Methods 2.1; Results 3.1

Section/Topic	Item	D/E	Checklist item	Reported on page
			participants with and without the outcome and, if applicable, a summary of the follow-up time. A diagram may be helpful	
	20b	D;E	Report the characteristics overall and, where applicable, for each data source or setting, including the key dates, key predictors (including demographics), treatments received, sample size, number of outcome events, follow-up time, and amount of missing data. A table may be helpful. Report any differences across key demographic groups	Table 1; Results 3.1
	20c	E	For model evaluation,	Not applicable (development

Section/Topic	Item	D/E	Checklist item	Reported on page
			show a comparison with the development data of the distribution of important predictors (demographics, predictors, and outcome)	study; site comparison in Table S2)
Model development	21	D;E	Specify the number of participants and outcome events in each analysis (e.g., for model development, hyperparameter tuning, model evaluation)	Results 3.1 (n=371, events=295, non-events=76)
Model specification	22	D	Provide details of the full prediction model (e.g., formula, code, object, application programming interface) to allow predictions in new individuals and to enable third-party evaluation and implementation, including	Table S10 (coefficients JSON); analysis code in File S2

Section/Topic	Item	D/E	Checklist item	Reported on page
			any restrictions to access or re-use	
Model performance	23a	D;E	Report model performance estimates with confidence intervals, including for any key subgroups (e.g., sociodemographic). Consider plots to aid presentation	Table 2; Figures 1–2; Results 3.3; Tables S5–S6
	23b	D;E	If examined, report results of any heterogeneity in model performance across clusters	Results 3.4; Table S8 (within-site and cross-site AUC)
Model updating	24	E	Report the results from any model updating, including the updated model and subsequent performance	Not applicable (development study)
**DISCUSSION**				
Interpretation	25	D;E	Give an overall interpretation of the main results,	Discussion paras 1–2

Section/Topic	Item	D/E	Checklist item	Reported on page
			including issues of fairness in the context of the objectives and previous studies	
Limitations	26	D;E	Discuss any limitations of the study (such as a non-representative sample, sample size, overfitting, missing data) and their effects on any biases, statistical uncertainty, and generalizability	Discussion paras 3–6
Usability	27a	D	Describe how poor quality or unavailable input data (e.g., predictor values) should be assessed and handled when implementing the prediction model	Not reported; training-fold median imputation described in Methods 2.4
	27b	D	Specify whether users will be required to	Not reported

Section/Topic	Item	D/E	Checklist item	Reported on page
			interact in the handling of the input data or use of the model, and what level of expertise is required of users	
	27c	D;E	Discuss any next steps for future research, with a specific view to applicability and generalizability of the model	Conclusions (harmonized gait-test settings, education-corrected cognitive scoring, independent validation)
